# Associations of negative affective biases and depressive symptoms in a community-based sample

**DOI:** 10.1017/S0033291722002720

**Published:** 2023-09

**Authors:** Laura de Nooij, Mark J. Adams, Emma L. Hawkins, Liana Romaniuk, Marcus R. Munafò, Ian S. Penton-Voak, Rebecca Elliott, Amy R. Bland, Gordon D. Waiter, Anca-Larisa Sandu, Tina Habota, J. Douglas Steele, Alison D. Murray, Archie Campbell, David J. Porteous, Generation Scotland, Andrew M. McIntosh, Heather C. Whalley

**Affiliations:** 1 Division of Psychiatry, University of Edinburgh, Edinburgh, UK; 2 Donders Institute for Brain, Cognition and Behaviour, Radboud University Medical Center, Nijmegen, The Netherlands; 3 Division of Imaging Science and Technology, School of Medicine, University of Dundee, Dundee, UK; 4 School of Psychological Science, University of Bristol, Bristol, UK; 5 MRC Integrative Epidemiology Unit, University of Bristol, Bristol, UK; 6 National Institute of Health Research Biomedical Research Centre, University Hospitals Bristol NHS Foundation Trust and the University of Bristol, Bristol, UK; 7 Division of Neuroscience & Experimental Psychology, University of Manchester, Manchester, UK; 8 Department of Psychology, Manchester Metropolitan University, Manchester, UK; 9 Aberdeen Biomedical Imaging Centre, Institute of Medical Sciences, University of Aberdeen, Aberdeen, UK; 10 Institute of Genetics and Cancer, University of Edinburgh, Edinburgh, UK

**Keywords:** Affective cognition, EMOTICOM, emotion recognition, facial processing, major depressive disorder, negative bias, remitted

## Abstract

**Background:**

Major depressive disorder (MDD) was previously associated with negative affective biases. Evidence from larger population-based studies, however, is lacking, including whether biases normalise with remission. We investigated associations between affective bias measures and depressive symptom severity across a large community-based sample, followed by examining differences between remitted individuals and controls.

**Methods:**

Participants from Generation Scotland (*N* = 1109) completed the: (i) Bristol Emotion Recognition Task (BERT), (ii) Face Affective Go/No-go (FAGN), and (iii) Cambridge Gambling Task (CGT). Individuals were classified as MDD-current (*n* = 43), MDD-remitted (*n* = 282), or controls (*n* = 784). Analyses included using affective bias summary measures (primary analyses), followed by detailed emotion/condition analyses of BERT and FAGN (secondary analyses).

**Results:**

For summary measures, the only significant finding was an association between greater symptoms and lower risk adjustment for CGT across the sample (individuals with greater symptoms were less likely to bet more, despite increasingly favourable conditions). This was no longer significant when controlling for non-affective cognition. No differences were found for remitted-MDD *v.* controls. Detailed analysis of BERT and FAGN indicated subtle negative biases across multiple measures of affective cognition with increasing symptom severity, that were independent of non-effective cognition [e.g. greater tendency to rate faces as angry (BERT), and lower accuracy for happy/neutral conditions (FAGN)]. Results for remitted-MDD were inconsistent.

**Conclusions:**

This suggests the presence of subtle negative affective biases at the level of emotion/condition in association with depressive symptoms across the sample, over and above those accounted for by non-affective cognition, with no evidence for affective biases in remitted individuals.

## Introduction

Many cognitive models of major depressive disorder (MDD) build on the classic cognitive theory (Beck, 1976); these newer models emphasise the role of both cognitive dysfunction and affective cognition biases in the aetiology and maintenance of depressive symptoms (LeMoult & Gotlib, 2019; Roiser & Sahakian, 2013). Previous research has indicated that MDD is associated with cognitive symptoms, particularly in the domains of executive functioning, memory, processing speed and attention (Lee, Hermens, Porter, & Redoblado-Hodge, 2012; Pantzar et al., 2017; Rock, Roiser, Riedel, & Blackwell, 2014), and suggests that these associations persist for remitted individuals (de Nooij et al., 2020). MDD is also associated with affective biases in perception, attention and memory (LeMoult & Gotlib, 2019; Roiser, Elliott, & Sahakian, 2012; Roiser & Sahakian, 2013). In contrast to never-depressed individuals, who typically show positive biases, individuals with MDD often show attenuated or negative biases (e.g. Duque & Vázquez, 2015; Erickson et al., 2005; Gollan, Pane, McCloskey, and Coccaro, 2008; Harmer et al., 2009). Previous studies have also investigated risk adjustment, a task measure which indicates to what extent risk-taking, in the context of gambling tasks, is adjusted in accordance with outcome probability. Individuals with MDD showed lower risk adjustment in response to positive reinforcement, potentially suggesting impaired reward motivation (Murphy et al., 2001; Rawal, Collishaw, Thapar, & Rice, 2013).

Studies of emotion recognition biases associated with depression were typically conducted using relatively small samples (*N* < 100) that were recruited using (clinical) case–control recruitment procedures. Although previous studies show converging evidence that MDD is associated with more negative affective biases, a wide variety of affective paradigms have been used, so that the type of biases reported – e.g. in relation to which particular emotions – often diverge between studies. Aiming to reduce paradigm heterogeneity, the EMOTICOM test battery was recently developed and validated for the assessment of affect-related cognition (Bland et al., 2016; Dam et al., 2019). This battery comprises variations and adaptations of commonly used affective cognition paradigms, as well as new paradigms. As such, EMOTICOM forms a standardised neuropsychological test battery to assess emotion processing, motivation, impulsivity, and social cognition – four core domains of affective cognition. This standardised EMOTICOM test battery could help identify the most prevailing types of affective biases associated with MDD.

Affective cognition differences in individuals remitted from MDD are less well understood than differences in currently depressed individuals. Some studies suggest biases may persist in individuals remitted from depression (Bhagwagar, Cowen, Goodwin, & Harmer, 2004; Fritzsche et al., 2010; LeMoult, Joormann, Sherdell, Wright, & Gotlib, 2009; Leppänen, 2006). In contrast, however, another study showed biases only for currently depressed, but not remitted individuals (Quigley, Wen, & Dobson, 2020). Cognitive models propose that negative biases may play a causal role in the onset and maintenance of depression (Ahern, Bockting, & Semkovska, 2019; Roiser & Sahakian, 2013). Cognitive bias modification (CBM) interventions target these negative biases to achieve remission (Jopling, Gotlib, & LeMoult, 2020; Lang, Blackwell, Harmer, Davison, & Holmes, 2012), which assumes that they characterise the depressive episode. The nature and extent of affective cognition differences in remitted individuals – particularly the manifestation within the general population – therefore remains unclear and warrants further investigation.

The purpose of the current study was to examine different types of affective cognition within a large community-based sample with and without lifetime MDD (a subsample of Generation Scotland) (Habota et al., 2019; Smith et al., 2013). Participants completed three affective cognition tasks: Bristol Emotion Recognition Task (BERT), Face Affective Go/No-Go (FAGN), and adapted Cambridge Gambling Task (CGT). We cross-sectionally investigated associations between depressive symptom severity [as measured by Quick Inventory of Depressive Symptomatology, QIDS, (Rush, Carmody, & Reimitz, 2000; Rush et al., 2003)] and abnormalities in affective cognition across the entire sample, as well as any differences in affective cognition for individuals remitted from depression relative to never-depressed individuals.

We hypothesised that depressive symptoms would be associated with differences in affective cognition and that these associations might be attenuated in remitted individuals. We initially focussed our analysis on the four affective cognitive summary measures, as highlighted by the developers of the EMOTICOM battery: summary measures of (i) BERT affective bias, (ii) FAGN affective bias, (iii) CGT risk adjustment win condition, and (iv) CGT risk adjustment loss condition. In addition, the richness of BERT and FAGN response data was investigated with more detailed statistical models of task conditions, to determine if there were differences in the patterns of biases beyond those conveyed by the summary measures. We hypothesised that depressive symptoms would be associated with a negative affective bias in emotion recognition (BERT), a negative affective bias in affective go/no-go response times (FAGN), and lower risk adjustment in the gambling task (CGT), indicative of impaired reward motivation (for win condition) and risk aversion (for loss condition). We initially tested relationships between depressive symptoms and affective cognition biases across the whole sample, and then examined differences between remitted MDD individuals (MDD-r) and controls. Follow-up analyses addressed whether any statistically significant findings were driven by non-affective cognition or subclinical symptoms, and explored effects related to antidepressant medication.

## Methods

### Participants

Participants were recruited as part of the Stratifying Resilience and Depression Longitudinally (STRADL) study cohort (Habota et al., 2019), which is a sub-cohort of the Scottish Family Health Study of Generation Scotland (GS:SFHS, described by Smith et al., 2013). Participants (*N* = 1179) attended one of two assessment centres at Dundee and Aberdeen Universities and completed affective cognition tasks. For the current analyses, we excluded participants with a history of a (hypo)mania, psychotic disorder, neurological disorder, or missing data linkage with Generation Scotland. The current sample therefore comprised *N* = 1109 individuals (*M*_age_ = 59.5 ± 10.0, age range = 26–84, 58.8% F). Sample demographic information is provided in Table 1. Ethics approval for all components of STRADL was obtained by approval from the NHS Tayside ethics committee (reference 14/SS/0039) and all participants provided written informed consent.

**Table 1.** Sample demographic information and affective cognition

	Control *n* = 784	MDD-r *n* = 282	MDD-c *n* = 43	*p* value
*Demographic information*				
*Age (years), median (IQR)*^a^	62 (7.3)	60 (10.8)	60 (7.3)	1.2 × 10^−8^ ^A^
*Sex female, n (%)*^b^	417 (53.2)	200 (70.9)	32 (74.4)	1.4 × 10^−7^ ^A^
*Education*^b^				0.22
Compulsory, *n* (%)	203 (25.9)	60 (21.3)	12 (27.9)	
> Compulsory, *n* (%)	262 (33.4)	111 (39.4)	18 (41.9)	
Post-secondary, *n* (%)	319 (40.7)	111 (39.4)	13 (30.2)	
*Handedness*^b^				0.63
Right, *n* (%)	701 (89.4)	250 (88.7)	41(95.3)	
Left, *n* (%)	56 (7.1)	19 (6.7)	1 (2.3)	
Mixed, *n* (%)	27 (3.4)	13 (4.6)	1 (2.3)	
*Total QIDS, median (IQR)*^a^	3 (2.0)	5 (4.0)	13 (6.0)	<2.2 × 10^−16^ ^B^
*Psychotropic medication*^c^, *n (%)*^b^	38 (4.8)	93 (33.0)	27 (62.8)	<2.2 × 10^−16^ ^B^
*Antidepressant medication, n (%*)^b^	30 (3.8)	85 (30.1)	26 (60.5)	<2.2 × 10^−16^ ^B^
*Affective cognition*				
*BERT, affective bias*				
*N*	773	282	42	
Mean (s.d.)^d^	0.04 (0.24)	0.05 (0.23)	0.05 (0.23)	0.86
*FAGN, affective bias*				
*N*	759	278	38	
Mean (s.d.)^d^	−0.03 (0.08)	−0.03 (0.06)	−0.02 (0.07)	0.65
*CGT, risk adjustment win*				
*N*	779	281	42	
Mean (s.d.)^d^	0.96 (1.28)	0.93(1.29)	0.96 (1.11)	0.97
*CGT, risk adjustment loss*				
*N*	778	281	42	
Mean (s.d.)^d^	1.10 (1.29)	1.09 (1.25)	1.33 (1.12)	0.51

BERT, Bristol Emotion Recognition Task; CGT, adapted Cambridge Gambling Task; FAGN, Face Go/No-Go; MDD-c, current major depressive disorder; MDD-r, remitted from major depressive disorder; RA, risk adjustment; QIDS, Quick Inventory of Depressive Symptomatology.

a Kruskal Wallis test, if significant followed up by Dunn's test for pairwise comparisons.

b Chi-squared test, if significant followed up by pairwise chi-squared tests.

c Use of antidepressant, antipsychotic, anticonvulsant, anxiolytic, hypnotic and/or mood-stabilising medications.

d One-way ANOVA test.

A Significant differences between control and MDD-r, and between control and MDD-c.

B Significant differences between all three groups.

### Materials

#### Clinical assessment

Lifetime occurrence of MDD, (hypo)mania and current diagnostic status were concurrently assessed with the Structural Clinical Interview for DSM-IV (SCID) (First, Spitzer, Gibbon, & Williams, 2002). For MDD, *n* = 43 individuals met the criteria for a current depressive episode (MDD-c). Remitted MDD (MDD-r) was defined by a history of one or more depressive episodes, without meeting current diagnostic criteria; this MDD-r group consisted of *n* = 282 individuals. The other *n* = 784 individuals had no history of MDD (controls). All study participants completed the Quick Inventory of Depressive Symptomatology (QIDS; range 0–27) to assess the severity of depressive symptoms (Rush et al., 2000, 2003).

#### Cognitive assessment

Affective cognition tasks were from the EMOTICOM test battery; for more information on the tasks and their evaluation, see Bland et al. (2016). The tasks were computer-based and administered in the following order: BERT, FAGN, CGT.

##### Bristol emotion recognition task (BERT)

Emotion recognition was assessed with the short version of BERT (Griffiths et al., 2017), which consists of a series of 96 faces displaying one of six emotions (anger, fear, happy, sad, surprise or disgust). Participants were asked to identify the displayed emotion with a forced choice between all six emotions. Faces were displayed on the screen for 300 ms (Griffiths et al., 2017), and were morphed to show emotions of eight different intensities, with every emotion displayed twice for each intensity (one male and one female face).

The summary outcome variable for the main analysis of the BERT was positive affective bias. This was calculated by subtracting the accuracy on sad trials from the accuracy on happy trials (Bland et al., 2016), hence larger values represent positive affective biases.

##### Face Go/No-Go (FAGN)

Information processing biases in facial expressions (happy, sad and neutral) were assessed with the FAGN (Bland et al., 2016). The task consists of six blocks that presented 20 faces each. Within each block, the participant was asked to respond by pressing the space key only in response to the target emotion (50%), needing to withhold responses for the distractor emotion. Here, a hit trial is a trial on which the target emotion is displayed, and the participant correctly responded with a button press.

The summary outcome variable for the main analysis for the FAGN was negative affective bias. This was determined by calculating the average of reaction times to happy/sad stimuli (‘happy’ target, ‘sad’ distractor) hit trials, minus the average reaction times to sad/happy stimuli (‘sad’ target, ‘happy’ distractor) hit trials (Bland et al., 2016). Higher values represent relatively slower response times to positive stimuli, which reflects negative biases.

##### Cambridge gambling task (CGT)

Decision-making and risk-taking were assessed with a modified version of the CGT (Bland et al., 2016). This version involves separate loss and win conditions, allowing the separation of reward and punishment. Participants were presented with a two-coloured roulette wheel on each trial. They were asked to select two chips of 5, 10 or 20 points each to bet one of the two colours. Proportions varied, so that the certainty of the bet ranged from very uncertain (50–50%) to very certain (90–10%). After betting, a spinning pointer landed on one colour and provided the participant with feedback on the outcome of the bet. The main outcome variables were risk adjustment, separately for the win condition (when the bet amount is doubled when winning) and loss condition (when the bet amount is lost when loosing).

Taking into account only the choices of the most likely outcomes, risk adjustment was calculated separately for each condition with the following formula: *risk adjustment* *=* (*2* *×* *bet at 90%*) *+* (*1* *×* *bet at 80%*) *+* (*0* *×* *bet at 70%*) *−* (*1* *×* *bet at 60%*) *−* (*2* *×* *bet at 50%*) */ average bet* (Bland et al., 2016). Higher risk adjustment indicates better adjustment of the bet according to the task win and loss probabilities (i.e. an increased total worth of the two chips for higher certainty bets).

##### Non-affective cognitive performance

In order to explore whether cognitive-affective differences could be accounted for by non-affective cognition deficits, a general factor of non-affective cognitive performance (*g*) was derived from five non-affective cognition tests also conducted at the same assessment as the affective tasks: (i) Matrix Reasoning (MR) (Ritchie et al., 1993), (ii) Verbal Fluency (VF), C-F-L (Lezak, 1995), (iii) Mill Hill Vocabulary test (MHV) (Raven, Court, & Raven, 1977), (iv) Logical Memory I story A (LM-story) immediate and delayed recall (Wechsler, 1998b), and (v) Digit Symbol Coding (DSC) (Wechsler, 1998a). After median imputation of missing data for DSC (*n* = 7), *g* was derived via principal component analysis of the five total correct test scores, followed by extraction of the first unrotated principal component. This principal component explained 41.7% of the variance. All five tests showed medium loading on *g* (0.40–0.47) (online Supplementary Table S1 in Supplemental Materials).

#### Data linkage

Because the cohort is family-based, information on family relatedness within the sample was retrieved via linkage with baseline data from the Generation Scotland study and controlled for in analysis as described below.

### Statistical analyses

All statistical analyses were done using R version 3.2.3. Coherence between the main affective cognition outcome variables and *g* was first investigated with a correlation matrix and hierarchical clustering (online Supplementary Figs S1 and S2 in Supplemental Materials). Subsequently, we tested for hypothesised associations between MDD and affective cognition using univariate generalised linear mixed models within the ‘MCMCglmm’ R package (Hadfield, 2010). This method uses the Markov Chain Monte Carlo algorithm, which is a Bayesian estimator for which we specified priors and model parameters that ensured convergence of models and mixing of multiple chains (for details see online Supplemental Materials). This method was selected to model data from this family-based cohort (taking into account familial relationships between participants), which in addition had non-Gaussian distributions for some outcome variables of interest (i.e. those which were not normally distributed).

All linear mixed models included age (transformed to *Z*-score) and sex as covariates and accounted for family-related variance by the implementation of a random effect. In our more detailed analyses, task conditions (e.g. emotion condition) were investigated with repeated measure models; these models included a random effect based on the subject-related variance added to the family-related variance.

Our statistical analyses consisted of Gaussian models for normally distributed outcome variables (standardised to *Z*-scores), including the summary measures, and logistic models for some of the detailed analyses as appropriate (for details see online Supplemental Materials). Effects were considered statistically significant if *p*MCMC < 0.05; there was no correction for multiple comparisons, as analyses were based on Bayesian estimators. To expand, in this modelling approach task conditions are jointly entered into a single model. Only one posterior distribution is therefore calculated (modelling all conditions), and all comparisons are made at the same time in the same model. In this type of modelling, there is no need to correct for multiple comparisons as these are all encompassed within a single model. We note also that the posterior distribution that is being sampled by the MCMCglmm algorithm is derived from the observed data, and is not necessarily a perfect, normal distribution. For Gaussian models, we report the mean of the standardised posterior distribution (*M*_posterior_), which is similar in interpretation to a standardised regression coefficient, along with its 95% ‘credible interval’ (95% CI). We note that ‘credible intervals’ are a Bayesian parallel of the typical ‘confidence interval’. These intervals show the range of parameters supported by the data and importantly do not change with the number of comparisons. For logistic models (i.e. family = ‘multinomial’ in ‘MCMCglmm’) we report the odds ratio (OR) and its 95% CI.

#### Primary analyses of EMOTICOM-based summary task outcomes

As discussed above, we initially considered the four main affective cognition outcome variables highlighted by Bland et al. (2016): (i) BERT affective bias, (ii) FAGN affective bias, (iii) CGT risk adjustment win condition, and then (iv) CGT risk adjustment loss condition. Four Gaussian models investigated associations with depressive symptoms (QIDS total score) across the whole sample, and were followed by four Gaussian models which investigated differences in affective cognition between MDD-r *v.* controls. The MDD-c group was not analysed as a separate group due to the limited sample size.

#### Secondary analyses of BERT and FAGN task conditions

Further analyses provided a more detailed investigation of the BERT and the FAGN affective cognition task response patterns associated with each MDD predictor, i.e. depressive symptoms (QIDS total score) or MDD-r *v.* controls. These MDD predictors were again investigated within separate models. We first studied the main effects related to depression severity averaged across conditions. For BERT, emotion recognition accuracy was aggregated over all six emotions. For FAGN, the hit/miss rate was calculated across all six conditions (hit-distractor combinations of emotions), as well as the false alarm rate and average reaction time on hit trials. We then conducted analyses with these measures aggregated across conditions to investigate patterns of overall lower performance on the tasks. Subsequently, we tested for emotion/condition interaction effects. These could indicate patterns of negative affective biases that are more subtle or complex, or between other task conditions than investigated with the summary affective bias measures. For BERT, outcome variables used in these analyses were accuracy of emotion recognition (i.e. correct selection of the emotion) and false alarm rate (i.e. erroneous selection of the emotion) per emotion. For FAGN, outcome variables used in these detailed analyses were hit/miss rate, false alarm rate, and average hit trial reaction time per condition. For more details see online Supplemental Materials.

#### Follow-up analyses

We also performed follow-up analyses for each statistically significant effect (for the affective cognition measures from primary or secondary analyses). We explored whether differences in affective cognition could be partially accounted for by differences in non-affective cognition (*g*). This included re-running previous models including *g* as a covariate of interest. To reduce the number of results presented, only the latter results for the detailed analysis of the BERT and FAGN task that remained significant after controlling for *g* are tabulated in the main body of the manuscript, all other results are detailed in the online Supplementary Materials. In addition, we also conducted two sensitivity analyses. We examined the effects of the antidepressant medication by in turn restricting our analysis to the medicated or medication-free MDD sample (for details see online Supplemental Materials). The potential effects of subclinical symptoms were also investigated for the comparison of MDD-r *v.* controls. Here, we examined the association between affective cognition and QIDS symptom scores while restricting our analysis to the MDD-r sample.

## Results

### Primary analyses of EMOTICOM-based summary outcomes

**Fig. 1.**
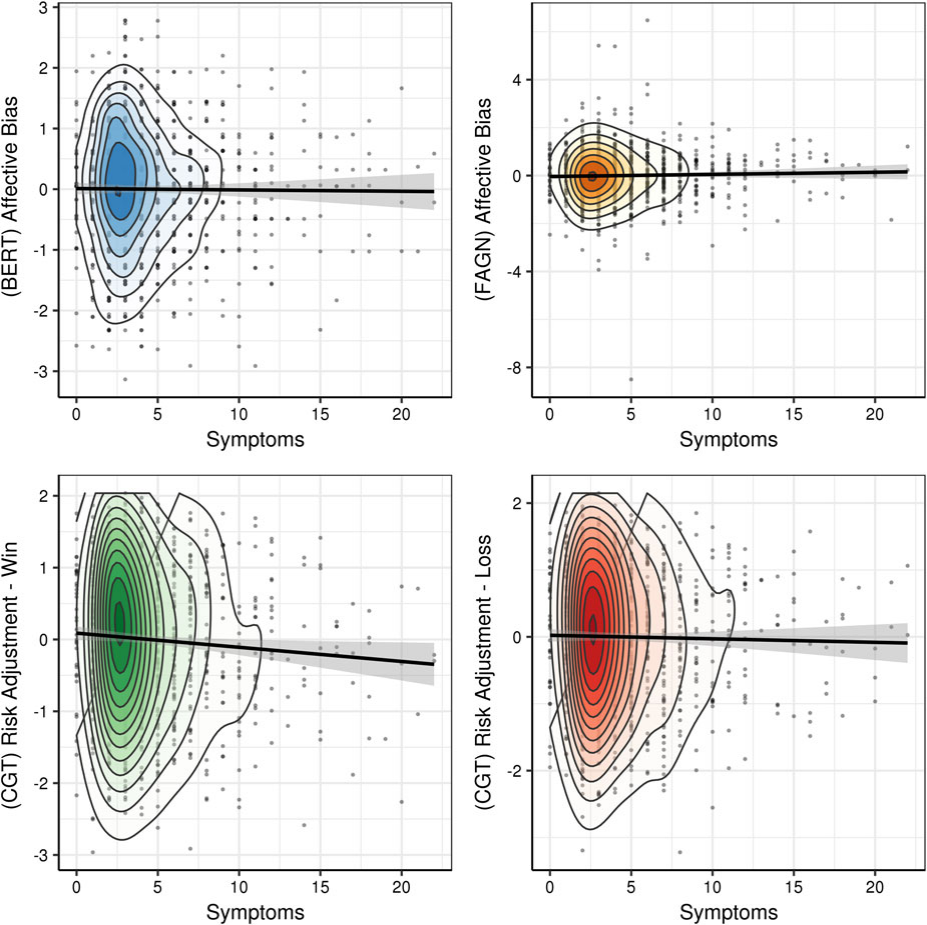
Main affective cognition outcome variables as a function of depression symptoms (QIDS score). Plots show minimal associations between symptoms scores and affective cognition measures, for which only the effect on CGT risk adjustment in the win condition was found to be marginally significant (*M*_posterior_ = −0.019, *p*MCMC = 0.03). Each outcome variable was normalised to a *Z*-score (*M* = 0, s.d. = 1), and for these plots, covariates age and sex were regressed out (using R function ‘lm’). The points represent observations, and are darker of colour when data points are overlapping. The circled contour layers represent the density of data points. A regression line was added based on a linear model (using R ggplot2 function ‘geom_smooth’) that predicted the outcome variable residuals from the QIDS symptoms score. BERT, Bristol Emotion Recognition Task; CGT, adapted Cambridge Gambling Task; FAGN, Face Go/No-Go; QIDS, Quick Inventory of Depressive Symptomatology.

**Fig. 2.**
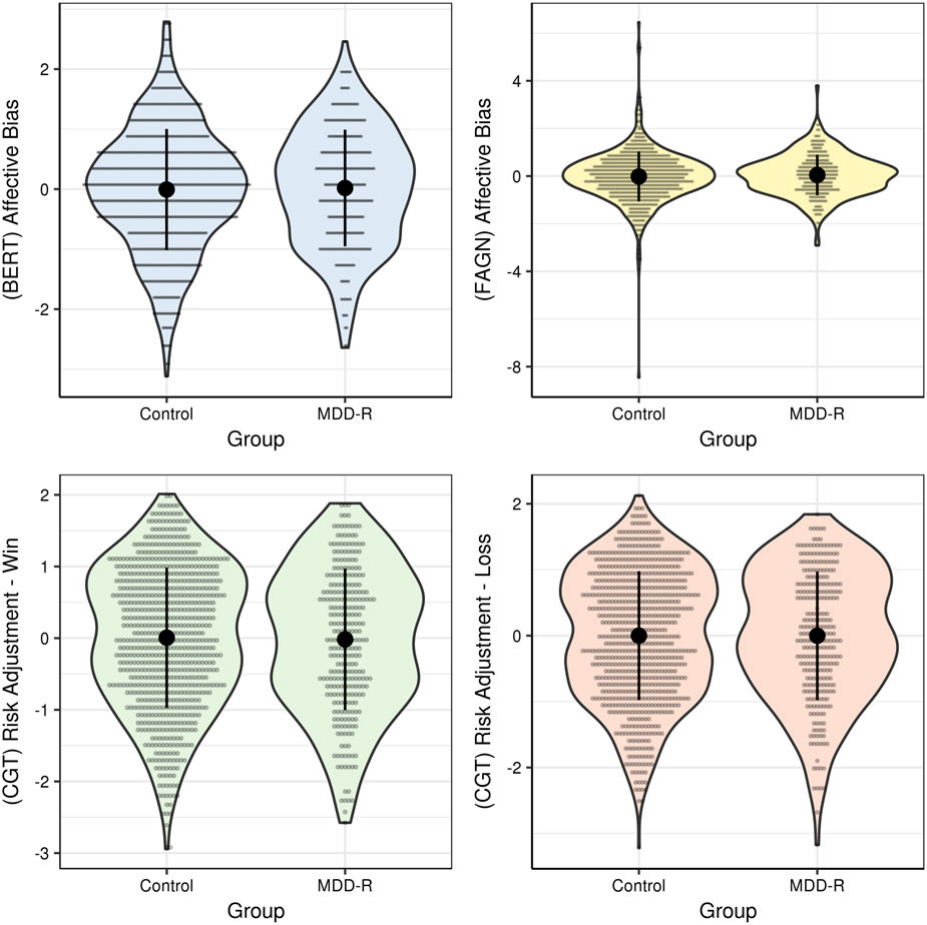
Differences in affective cognition outcome variables related to remitted depression. Each outcome variable was normalised to a *Z*-score (*M* = 0, s.d. = 1) and covariates age and sex were regressed out (using R function ‘lm’). The plots show differences in each outcome measure for MDD-r *v.* controls. The violin plots reflect the variable distributions per group. Data points (one for each observation) were vertically binned and horizontally stacked, appearing as horizontal lines. Means and standard deviations (per group) are indicated by the black points and vertical lines. BERT, Bristol Emotion Recognition Task; CGT, adapted Cambridge Gambling Task; FAGN, Face Go/No-Go; MDD-r, individuals remitted from major depressive disorder.

Descriptive statistics for the four main EMOTICOM-based summary outcome variables did not differ between MDD-c, MDD-r and controls (Table 1). Associations of the EMOTICOM-based outcomes with depressive symptoms, and differences between MDD-r *v.* controls, respectively, are shown in Table 2 and Figs 1 and 2. Regarding depressive symptoms, the results showed a significant association with the CGT risk adjustment win condition (*M*_posterior_ = −0.02, 95% CI −0.04 to 0.00, *p*MCMC = 0.03). This indicated lower risk adjustment to outcome probability when gambling for monetary reinforcement in relation to increasing depressive symptoms across the sample. This effect was attenuated when *g* was included as covariate of interest (59% remaining; *M*_posterior_ = −0.01, 95% CI −0.03 to 0.01, *p*MCMC = 0.17). The sensitivity analysis indicated this effect was significant when restricting the analysis to individuals taking antidepressants (*M*_posterior_ = −0.05, 95% CI −0.09 to −0.02, *p*MCMC = 0.004), but not when restricting to individuals *not* taking antidepressants (*M*_posterior_ = 0.02, 95% CI −0.02 to 0.05, *p*MCMC = 0.42). There was no clear evidence of an association between depressive symptom severity and summary measures BERT affective bias, FAGN affective bias, and CGT risk adjustment loss condition, nor for differences between MDD-r and controls (Table 2).

**Table 2.** Results of primary analyses: associations between MDD and EMOTICOM summary affective cognition outcome variables

	*M*_posterior_	95% CI	*p*MCMC
Symptoms			
BERT, affective bias	−0.002	[−0.019 to 0.014]	0.79
FAGN, affective bias	0.007	[−0.009 to 0.026]	0.45
CGT, risk adjustment win	−0.019	[−0.038 to −0.003]	0.03*
CGT, risk adjustment loss	−0.005	[−0.023 to 0.010]	0.54
MDD-r *v.* Control			
BERT, affective bias	0.027	[−0.120 to 0.159]	0.70
FAGN, affective bias	0.059	[−0.084 to 0.201]	0.41
CGT, risk adjustment win	−0.022	[−0.181 to 0.106]	0.76
CGT, risk adjustment loss	−0.005	[−0.149 to 0.131]	0.96

BERT, Bristol Emotion Recognition Task; CGT, adapted Cambridge Gambling Task; CI, credible interval; FAGN, Face Go/No-Go; MCMC, Markov Chain Monte Carlo; MDD-r, remitted from major depressive disorder.

*pMCMC < 0.05.

### Secondary analyses of BERT and FAGN task conditions

The more detailed analysis of these tasks, including sensitivity analyses, are reported in full in Supplemental Materials (online Supplementary Figs S3 and S4, Tables S2–S11). For brevity, we report here only statistically significant findings, together with follow-up analyses that included *g* as a covariate. Sensitivity analyses of antidepressant medication are reported in Supplemental Materials (online Supplementary Tables S4 and S10–S11).

#### General effects on cognitive-affective performance (across conditions) across sample

When aggregating performance measures across all emotions/conditions, findings indicated that increasing depressive symptom severity was significantly associated with lower BERT emotion recognition accuracy (OR 0.99, 95% CI 0.99 to 1.00, *p*MCMC = 0.01), lower FAGN hit rate (OR 0.96, 95% CI 0.95 to 0.98, *p*MCMC < 0.001), increased FAGN false alarms (OR 0.99, 95% CI 0.97 to 1.00, *p*MCMC = 0.05) and slower FAGN reaction times (*M*_posterior_ = 0.03, 95% CI [0.02–0.05], *p*MCMC < 0.001), online Supplementary Table S2. When including *g* as a covariate, only the effects of lower FAGN hit rate and higher FAGN reaction time remained statistically significant (FAGN hit rate, OR −0.02, 95% CI 0.99 to 1.00, *p*MCMC = 0.01; FAGN reaction time, *M*_posterior_ = 0.03, 95% CI 0.01 to 0.05, *p*MCMC < 0.001), indicating a generally slower and less accurate response pattern (independent of general non-affective cognitive ability), Table 3.

**Table 3.** Results of secondary analyses controlling for cognitive ability (*g*) in statistically significant associations between depressive symptoms and affective cognitive performance measures aggregated over all emotions/conditions

BERT, accuracy	*M*_posterior_	%effect	OR	OR 95% CI	*p*MCMC
*g*	0.141		1.15	[1.13 to 1.17]	<0.001***
Symptoms	−0.002	24%	1.00	[0.99 to 1.00]	0.522
FAGN, hit rate	*M*_posterior_	%effect	OR	OR 95% CI	*p*MCMC
*g*	0.324		1.38	[1.30 to 1.48]	<0.001***
Symptoms	−0.022	60%	0.98	[0.96 to 1.00]	0.020*
FAGN, false alarms	*M*_posterior_	%effect	OR	OR 95% CI	*p*MCMC
*g*	0.187		1.21	[1.15 to 1.26]	<0.001***
Symptoms	−0.007	46%	0.99	[0.98 to 1.01]	0.286
FAGN, reaction time	*M*_posterior_	%effect		95% CI	*p*MCMC
*g*	−0.077			[−0.14 to −0.02]	0.020*
Symptoms	0.031	90%		[0.01 to 0.05]	<0.001***

BERT, Bristol Emotion Recognition Task; CI, credible interval; FAGN, Face Go/No-Go; *g*, general factor of cognitive ability; MCMC, Markov Chain Monte Carlo; OR, odds ratio.

*pMCMC < 0.05, ***pMCMC < 0.001.

#### Affective biases associated with depressive symptoms (by condition) across sample

Taking conditions separately, for BERT, depressive symptoms were associated with a higher emotion recognition accuracy of angry relative to happy faces (OR 1.03, 95% CI 1.01 to 1.05], *p*MCMC < 0.001), online Supplementary Table S5. Furthermore, depressive symptom severity was associated with an increased number of false alarms, where faces were incorrectly recognised as angry (OR 0.96, 95% CI 0.94 to 0.98, *p*MCMC < 0.001) or as disgust (OR 0.98, 95% CI 0.96 to 0.99], *p*MCMC = 0.008) compared to the happy reference condition.

Within FAGN models, depressive symptoms were associated with lower hit rates in the happy/neutral reference condition (OR 0.93, 95% CI 0.89 to 0.98], *p*MCMC = 0.004). Increasing depressive symptom severity was also associated with faster reaction times in the neutral/happy condition (*M*_posterior_ = −0.03, 95% CI −0.04 to −0.01, *p*MCMC = 0.002) and the sad/neutral condition (*M*_posterior_ = −0.02, 95% CI −0.04 to −0.01 compared to the happy/neutral reference condition, *p*MCMC = 0.02.

The negative affective biases reported above were not attenuated in follow-up analyses that included *g* as a covariate of interest, see Table 4, even though depressive symptoms were associated with lower *g* (*M*_posterior_ = −0.05, 95% CI −0.06 to −0.03, *p*MCMC < 0.001).

**Table 4.** Results of detailed analysis analyses controlling for cognitive ability (*g*) in statistically significant emotion/condition-specific associations between depressive symptoms and affective cognitive performance

BERT, accuracy	*M*_posterior_	%effect	OR 95% CI	*p*MCMC
*g*	0.166		[1.15 to 1.21]	<0.001***
Symptoms (ref. Happy)	−0.012	62%	[0.97 to 1.00]	0.104
Symptoms:Angry	0.025	99%	[1.01 to 1.04]	<0.001***
BERT, false alarms	*M*_posterior_	%effect	OR 95% CI	*p*MCMC
*g*	0.087		[1.07 to 1.11]	<0.001***
Symptoms:Angry	−0.038	99%	[0.95 to 0.98]	<0.001***
Symptoms:Disgust	−0.023	98%	[0.96 to 0.99]	0.004**
FAGN, hit rate	*M*_posterior_	%effect	OR 95% CI	*p*MCMC
*g*	0.393		[1.38 to 1.60]	<0.001***
Symptoms (ref. Happy/neutral)	−0.055	76%	[0.90 to 0.99]	0.018*
FAGN, reaction time	*M*_posterior_	%effect	95% CI	*p*MCMC
*g*	−0.031		[−0.08 to 0.01]	0.160
Symptoms:Neutral/happy	−0.026	101%	[−0.04 to −0.01]	0.002**
Symptoms:Sad/neutral	−0.020	98%	[−0.04 to −0.01]	0.018*

BERT, Bristol Emotion Recognition Task; CI, credible interval; FAGN, Face Go/No-Go; *g*, general factor of cognitive ability; MCMC, Markov Chain Monte Carlo; OR, odds ratio; ref., reference emotion/condition.

*pMCMC < 0.05, **pMCMC < 0.01, ***pMCMC < 0.001.

#### Affective biases associated with remitted MDD

BERT analyses indicated that MDD-r showed a relatively decreased number of false alarms in which faces were incorrectly identified as sad, compared to false alarms on the happy reference emotion (OR 1.16, 95% CI 1.00 to 1.32], *p*MCMC = 0.03), see online Supplementary Table S8. FAGN analyses showed a decreased false alarm rate in the sad/neutral condition (OR 1.33, 95% CI 1.02 to 1.70, *p*MCMC = 0.04) for MDD-r *v.* controls. Both of these effects potentially reflect positive biases. In contrast, however, a negative bias was indicated by a faster FAGN reaction time in the sad/happy condition (*M*_posterior_ = −0.10, 95% CI −0.27 to 0.00, *p*MCMC = 0.05) relative to the happy/neutral reference condition. The above effects remained after covarying for *g*,a measure that also did not reliably differ between groups (*M*_posterior_ = −0.04, 95% CI −0.17 to 0.09, *p*MCMC = 0.55).

## Discussion

We investigated negative affective biases associated with depressive symptoms, and whether these biases were present within individuals remitted from MDD. Primary analyses of EMOTICOM-based summary task outcomes addressed four affective cognition outcome measures from three paradigms (BERT, FAGN, CGT) highlighted by EMOTICOM (Bland et al., 2016). The results indicated that current depressive symptoms across the sample were significantly associated with lower CGT risk adjustment in the win condition. This suggests reduced motivation for rewards, as in those with increased depressive symptoms were less likely to bet more points under increasingly favourable conditions compared to those with lower depression scores. Although this corresponds to previous findings of decreased responsiveness to rewards in depression (Henriques & Davidson, 2000; Whitton, Treadway, & Pizzagalli, 2015), the current finding was however largely attenuated when accounting for non-affective cognitive performance (*g*). This corroborates cognitive models that propose a close link between affective and non-affective cognition (Ahern et al., 2019; Roiser & Sahakian, 2013) and emphasise that many affective paradigms also require (non-affective) cognitive effort. In addition, sensitivity analysis indicated this effect was statistically significant when restricting the analysis to individuals taking antidepressants, but not when restricting to individuals who were medication-free. No evidence was found for other affective cognition differences with increasing symptom severity using the remaining EMOTICOM-based summary task outcomes, or in remitted MDD *v.* controls.

Since the analysis of the EMOTICOM-based summary task outcomes may miss some more fine-grained patterns of potential cognition biases, we additionally performed secondary analyses that were more detailed analyses of BERT and FAGN task conditions to identify subtle effects per emotion or condition. We found increasing depressive symptom severity across the whole sample was associated with increased accuracy on the BERT task for angry faces, and an increased number of false alarms, where faces were incorrectly recognised as displaying anger or disgust compared to happiness. For the FAGN, increasing depressive symptom severity was associated with negative biases including lower hit rates in the happy/neutral reference condition and faster reaction times in the neutral/happy condition and the sad/neutral condition. Although this is in line with previous findings of increased attentional biases towards angry faces (Leyman, De Raedt, Schacht, & Koster, 2007), previous meta-analyses of facial emotion recognition impairments related to MDD did not show relatively higher biases for angry faces (Dalili, Penton-Voak, Harmer, & Munafò, 2015; Krause, Linardatos, Fresco, & Moore, 2021). We note that while these analyses require replication, they highlight the consistency of significant effects in relation to depressive symptoms. These results suggest subtle negative biases for the interpretation of emotional faces associated with depression symptoms within this population-based sample.

In contrast, the detailed analysis findings for remitted MDD *v.* controls showed few statistically significant differences in affective cognition. Furthermore, the interpretation of these differences is less straightforward due to inconsistent directions of biases (i.e. both positive and negative biases). Overall, our study shows limited evidence for the persistence of negative biases in individuals remitted from MDD.

The present study has a number of strengths. Most importantly, the STRADL cohort (*N* > 1000), a subsample of Generation Scotland, is community-based and provides a substantially greater sample size than most previous studies (generally, *N* < 100). Of note, the scale of the study did not compromise the quality of phenotypic assessments such as mood disorder classification, which was reliably assessed via a structured clinical interview. The generous sample size also allowed more detailed analyses of affective cognitive performance by complex modelling, revealing subtle patterns of negative biases. Notably, the ‘MCMCglmm’ algorithm modelled the joint posterior distribution of each outcome variable based on all different predictor variable levels while adjusting for family-related variance all in the same model. In this type of modelling, there is no need to correct for multiple comparisons as these are all encompassed within a single model, which contrasts with other (frequentist) approaches (Gelman, Hill, & Yajima, 2012).

Limitations include the smaller number of currently depressed individuals, resulting in a lack of power for a proper investigation; we therefore decided to not explore affective cognition differences related to current MDD status. Further, we note that the effect sizes reported here were relatively small, as is often the case with large community-based population studies, limiting the clinical utility of these findings. However, these findings do represent a consistent pattern of relationships between subtle affective biases and the severity of depressive symptoms across the whole population studied. Results are also only limited to three affective paradigms, whereas other types of negative cognitive biases have also been reported (e.g. see full EMOTICOM test battery). Furthermore, this study was of cross-sectional nature and investigated group-level effects. Medication effects were explored, but it is difficult to separate these from clinical characteristics within cross-sectional and correlational study designs, nor was it possible to determine whether effects were a result of the medication itself or the clinical characteristics of the group. Consequently, effects related to antidepressant use may be driven by other characteristics such as more severe symptomatology, recurrence, or comorbidity.

In summary, across the whole sample, we report that individuals with higher current depressive symptoms show a reduced motivation for reward, however, this was partially accounted for by non-affective cognition and antidepressant use. In contrast, in the detailed secondary analyses, there was consistent evidence for subtle facial affective processing biases in association with increased depressive symptom severity, which remained after controlling for non-affective cognition. Future research may investigate other domains of affective processing and their association with current MDD. Notably, studies of longitudinal differences in affective cognitive performance over time at an individual level would allow for a more detailed investigation into these effects.

## Supplementary material

For supplementary material accompanying this paper visit https://doi.org/10.1017/S0033291722002720. click here to view supplementary material

## References

[ref1] Ahern, E., Bockting, C. L. H., & Semkovska, M. (2019). A hot-cold cognitive model of depression: Integrating the neuropsychological approach into the cognitive theory framework. Clinical Psychology in Europe, 1(3), e34396. 10.32872/cpe.v1i3.34396

[ref2] Beck, A. T. (1976). Cognitive therapy and the emotional disorders. Oxford, UK: International Universities Press.

[ref3] Bhagwagar, Z., Cowen, P. J., Goodwin, G. M., & Harmer, C. J. (2004). Normalization of enhanced fear recognition by acute SSRI treatment in subjects with a previous history of depression. American Journal of Psychiatry, 161, 166–168. 10.1176/appi.ajp.161.1.166. 14702268

[ref4] Bland, A. R., Roiser, J. P., Mehta, M. A., Schei, T., Boland, H., Campbell-Meiklejohn, D. K., … Elliott, R. (2016). EMOTICOM: A neuropsychological test battery to evaluate emotion, motivation, impulsivity, and social cognition. Frontiers in Behavioral Neuroscience, 10, 25. 10.3389/fnbeh.2016.00025. 26941628 PMC4764711

[ref5] Dalili, M. N., Penton-Voak, I. S., Harmer, C. J., & Munafò, M. R. (2015). Meta-analysis of emotion recognition deficits in major depressive disorder. Psychological Medicine, 45, 1135–1144. 10.1017/S0033291714002591. 25395075 PMC4712476

[ref6] Dam, V. H., Thystrup, C. K., Jensen, P. S., Bland, A. R., Mortensen, E. L., Elliott, R., … Stenbæk, D. S. (2019). Psychometric properties and validation of the EMOTICOM test battery in a healthy Danish population. Frontiers in Psychology, 10, 1–15. 10.3389/fpsyg.2019.02660. 31849772 PMC6901831

[ref7] de Nooij, L., Harris, M. A., Adams, M. J., Clarke, T.-K., Shen, X., Cox, S. R., … Whalley, H. C. (2020). Cognitive functioning and lifetime major depressive disorder in UK Biobank. European Psychiatry, 63(1). 10.1192/j.eurpsy.2020.24. PMC7315876 32189608

[ref8] Duque, A., & Vázquez, C. (2015). Double attention bias for positive and negative emotional faces in clinical depression: Evidence from an eye-tracking study. Journal of Behavior Therapy and Experimental Psychiatry, 46, 107–114. 10.1016/j.jbtep.2014.09.005. 25305417

[ref9] Erickson, K., Drevets, W. C., Clark, L., Cannon, D. M., Bain, E. E., Zarate, C. A., … Sahakian, B. J. (2005). Mood-congruent bias in affective go/no-go performance of unmedicated patients with major depressive disorder. American Journal of Psychiatry, 162, 2171–2173. 10.1176/appi.ajp.162.11.2171. 16263859

[ref10] First, M. B., Spitzer, R. L., Gibbon, M., & Williams, J. B. (2002). Structured clinical interview for DSM-IV-TR axis I disorders, research version, patient edition with psychotic screen. New York: Biometrics Research, New York State Psychiatric Institute.

[ref11] Fritzsche, A., Dahme, B., Gotlib, I. H., Joormann, J., Magnussen, H., Watz, H., … von Leupoldt, A. (2010). Specificity of cognitive biases in patients with current depression and remitted depression and in patients with asthma. Psychological Medicine, 40, 815–826. 10.1017/S0033291709990948. 19719897 PMC2847035

[ref12] Gelman, A., Hill, J., & Yajima, M. (2012). Why we (usually) don't have to worry about multiple comparisons. Journal of Research on Educational Effectiveness, 5, 189–211. 10.1080/19345747.2011.618213.

[ref13] Gollan, J. K., Pane, H. T., McCloskey, M. S., & Coccaro, E. F. (2008). Identifying differences in biased affective information processing in major depression. Psychiatry Research, 159, 18–24. 10.1016/j.psychres.2007.06.011. 18342954 PMC2571942

[ref14] Griffiths, S., Jarrold, C., Penton, I. S., Andy, V., Skinner, A. L., & Munafò, M. R. (2017). Impaired recognition of basic emotions from facial expressions in young people with autism spectrum disorder: Assessing the importance of expression intensity. Journal of Autism and Developmental Disorders, *49*, 2768–2778. 10.1007/s10803-017-3091-7. PMC6606653 28361375

[ref15] Habota, T., Sandu, A.-L., Waiter, G. D., McNeil, C. J., Steele, J. D., Macfarlane, J. A., … McIntosh, A. M. (2019). Cohort profile: Stratifying resilience and depression longitudinally (STRADL): A depression-focused investigation of Generation Scotland, using detailed clinical, cognitive, and neuroimaging assessments. Wellcome Open Research, 4, 184. 10.12688/wellcomeopenres.15538.1. 35237729 PMC8857525

[ref16] Hadfield, J. D. (2010). MCMC methods for multi-response generalized linear mixed models: The MCMCglmm R package. Journal of Statistical Software, 33(2), 1–22. 10.18637/jss.v033.i02.20808728

[ref17] Harmer, C. J., O'Sullivan, U., Favaron, E., Massey-Chase, R., Ayres, R., Reinecke, A., … Cowen, P. J. (2009). Effect of acute antidepressant administration on negative affective bias in depressed patients. American Journal of Psychiatry, 166, 1178–1184. 10.1176/appi.ajp.2009.09020149. 19755572

[ref18] Henriques, J. B., & Davidson, R. J. (2000). Decreased responsiveness to reward in depression. Cognition and Emotion, 14(5), 711–724. 10.1080/02699930050117684.

[ref19] Jopling, E., Gotlib, I. H., & LeMoult, J. (2020). Effects of working memory training on cognitive, affective, and biological responses to stress in major depression: A novel cognitive bias modification protocol. Journal of Affective Disorders, 265, 45–51. 10.1016/j.jad.2020.01.007. 31957691 PMC7050386

[ref20] Krause, F. C., Linardatos, E., Fresco, D. M., & Moore, M. T. (2021). Facial emotion recognition in major depressive disorder: A meta-analytic review. Journal of Affective Disorders, 293, 320–328. 10.1016/j.jad.2021.06.053. 34229285 PMC8457509

[ref21] Lang, T. J., Blackwell, S. E., Harmer, C. J., Davison, P., & Holmes, E. A. (2012). Cognitive bias modification using mental imagery for depression: Developing a novel computerized intervention to change negative thinking styles. European Journal of Personality, 26, 145–157. 10.1002/per. 23316101 PMC3532611

[ref22] Lee, R. S. C., Hermens, D. F., Porter, M. A., & Redoblado-Hodge, M. A. (2012). A meta-analysis of cognitive deficits in first-episode major depressive disorder. Journal of Affective Disorders, 140, 113–124. 10.1016/j.jad.2011.10.023. 22088608

[ref23] LeMoult, J., & Gotlib, I. H. (2019). Depression: A cognitive perspective. Clinical Psychology Review, 69, 51–66. 10.1016/j.cpr.2018.06.008. 29961601 PMC11884012

[ref24] LeMoult, J., Joormann, J., Sherdell, L., Wright, Y., & Gotlib, I. H. (2009). Identification of emotional facial expressions following recovery from depression. Journal of Abnormal Psychology, 118(4), 828–833. 10.1037/a0016944. 19899852 PMC2837802

[ref25] Leppänen, J. M. (2006). Emotional information processing in mood disorders: A review of behavioral and neuroimaging findings. Current Opinion in Psychiatry, 19, 34–39. 10.1097/01.yco.0000191500.46411.00. 16612176

[ref26] Leyman, L., De Raedt, R., Schacht, R., & Koster, E. H. W. (2007). Attentional biases for angry faces in unipolar depression. Psychological Medicine, 37(3), 393–402. 10.1017/S003329170600910X. 17076914

[ref27] Lezak, M. D. (1995). Neuropsychological testing (3rd ed.). Oxford: Oxford University Press.

[ref28] Murphy, F. C., Rubinsztein, J. S., Michael, A., Rogers, R. D., Robbins, T. W., Paykel, E. S., … Sahakian, B. J. (2001). Decision-making cognition in mania and depression. Psychological Medicine, *31*, 679–693. 10.1017/s0033291701003804. 11352370

[ref29] Pantzar, A., Atti, A. R., Fratiglioni, L., Fastbom, J., Bäckman, L., & Laukka, E. J. (2017). Cognitive performance in unipolar old-age depression: A longitudinal study. International Journal of Geriatric Psychiatry, 32, 675–684. 10.1002/gps.4510. 27246314

[ref30] Quigley, L., Wen, A., & Dobson, K. S. (2020). Cognitive control over emotional information in current and remitted depression. Behaviour Research and Therapy, 132, 103658. 10.1016/j.brat.2020.103658. 32615318

[ref31] Raven, J. C., Court, J. H., & Raven, J. (1977). Manual for Raven's progressive matrices and vocabulary scales (H. K. Lewis, Ed.). London: H. K. Lewis.

[ref32] Rawal, A., Collishaw, S., Thapar, A., & Rice, F. (2013). ‘The risks of playing it safe’: A prospective longitudinal study of response to reward in the adolescent offspring of depressed parents. Psychological Medicine, 43, 27–38. 10.1017/S0033291712001158. 22617461

[ref33] Ritchie, K., Allard, M., Huppert, F. A., Nargeot, C., Pinek, B., & Ledesert, B. (1993). Computerized cognitive examination of the elderly (ECO): The development of a neuropsychological examination for clinic and population use. International Journal of Geriatric Psychiatry, 8, 899–914. 10.1002/gps.930081104.

[ref34] Rock, P. L., Roiser, J. P., Riedel, W. J., & Blackwell, A. D. (2014). Cognitive impairment in depression: A systematic review and meta-analysis. Psychological Medicine, 44, 2029–2040. 10.1017/S0033291713002535. 24168753

[ref35] Roiser, J. P., Elliott, R., & Sahakian, B. J. (2012). Cognitive mechanisms of treatment in depression. Neuropsychopharmacology Reviews, 37, 117–136. 10.1038/npp.2011.183. 21976044 PMC3238070

[ref36] Roiser, J. P., & Sahakian, B. J. (2013). Hot and cold cognition in depression. CNS Spectrums, 18, 139–149. 10.1017/S1092852913000072. 23481353

[ref37] Rush, A. J., Carmody, T., & Reimitz, P.-E. (2000). The inventory of depressive symptomatology (IDS): Clinician (IDS-C) and self-report (IDS-SR) ratings of depressive symptoms. International Journal of Methods in Psychiatric Research, 9(2), 45–59. 10.1002/mpr.79.

[ref38] Rush, A. J., Trivedi, M. H., Ibrahim, H. M., Carmody, T. J., Arnow, B., Klein, D. N., … Keller, M. B. (2003). The 16-item quick inventory of depressive symptomatology (QIDS), clinical rating (QIDS-C), and self-report (QIDS-SR): A psychometric evaluation in patients with chronic Major depression. Biological Psychiatry, 54, 573–583. 10.1016/S0006-3223(03)01866-8. 12946886

[ref39] Smith, B. H., Campbell, A., Linksted, P., Fitzpatrick, B., Jackson, C., Kerr, S. M., … Morris, A. D. (2013). Cohort profile: Generation Scotland: Scottish family health study (GS:SFHS). The study, its participants and their potential for genetic research on health and illness. International Journal of Epidemiology, 42, 689–700. 10.1093/ije/dys084. 22786799

[ref40] Wechsler, D. (1998a). Wechsler adult intelligence scale III UK. London: Psychological Corporation.

[ref41] Wechsler, D. (1998b). Wechsler memory scale III UK. London: Psychological Corporation.

[ref42] Whitton, A. E., Treadway, M. T., & Pizzagalli, D. A. (2015). Reward processing dysfunction in major depressive disorder, bipolar disorder and schizophrenia. Current Opinion in Psychiatry, 28(1), 7–12. 10.1097/YCO.0000000000000122. 25415499 PMC4277233

