# Supplementary material for: Associations of negative affective biases and depressive symptoms in a community-based sample
Source: Psychol Med. 2022 Sep 21;53(12):5518–27. doi: 10.1017/S0033291722002720 (PMC10482721; doi:10.1017/S0033291722002720)
Supplement: Supplementary file 1 [file S0033291722002720sup001.pdf]

Supplemental Materials

Associations of negative affective biases and depressive symptoms in a community-based sample

CONTENTS

- **Table S1**: Principal Component loadings on cognitive ability (*g*)
- **Figure S1**: Pearson correlations between summary affective cognition outcome variables and *g*
  Figure S2: Hierarchical relationships between summary affective cognition outcome variables and *g*
- **Statistical analysis: information on weak priors and model parameters**
- **Statistical analysis: detailed analyses (per task)**
- **Statistical analysis: sensitivity analyses regarding use of antidepressant medication**
- **Figure S3**: BERT response patterns across the entire sample
- **Figure S4**: FAGN response patterns across the entire sample
- **Table S2**: Results of detailed analyses that investigated main effects of depressive symptoms and remitted depression by aggregating affective cognitive performance measures over emotions/conditions
- **Table S3**: Results of follow-up analyses regarding the role of cognitive ability (*g*) in statistically significant associations between depressive symptoms and affective cognitive performance measures aggregated over emotions/conditions
- **Table S4**: Results of sensitivity analyses regarding use of antidepressant medication for statistically significant associations between depressive symptoms and affective cognitive performance measures aggregated over emotions/conditions
- **Table S5**: Results of detailed analyses regarding emotion/condition-specific associations between depressive symptoms and affective cognitive performance
- **Table S6**: Results of detailed analyses regarding emotion/condition-specific associations between remitted depression and affective cognition
- **Table S7**: Results of follow-up analyses regarding the role of cognitive ability (*g*) in statistically significant emotion/condition-specific associations between depressive symptoms and affective cognition
- **Table S8**: Results of follow-up analyses regarding the role of cognitive ability (*g*) in statistically significant emotion/condition-specific associations between remitted depression and affective cognition

- **Table S9**: Results of sensitivity analyses regarding subclinical symptoms for statistically significant emotion/condition-specific associations between remitted depression and affective cognition
- **Table S10**: Results of sensitivity analyses regarding use of antidepressant medication for statistically significant emotion/condition-specific associations between depressive symptoms and affective cognition
- **Table S11**: Results of sensitivity analyses regarding use of antidepressant medication for statistically significant emotion/condition-specific associations between remitted depression and affective cognition
- **Additional information**

Table S1. Principal Component loadings on cognitive ability (*g*)

**Non-affective cognitive test**	**Loading**
Matrix Reasoning (MR)	0.472
Verbal Fluency, C-F-L (VF)	0.454
Mill Hill Vocabulary test (MHV)	0.475
Logical Memory I Story A (LM-story)	0.428
Digit Symbol Coding (DSC)	0.403

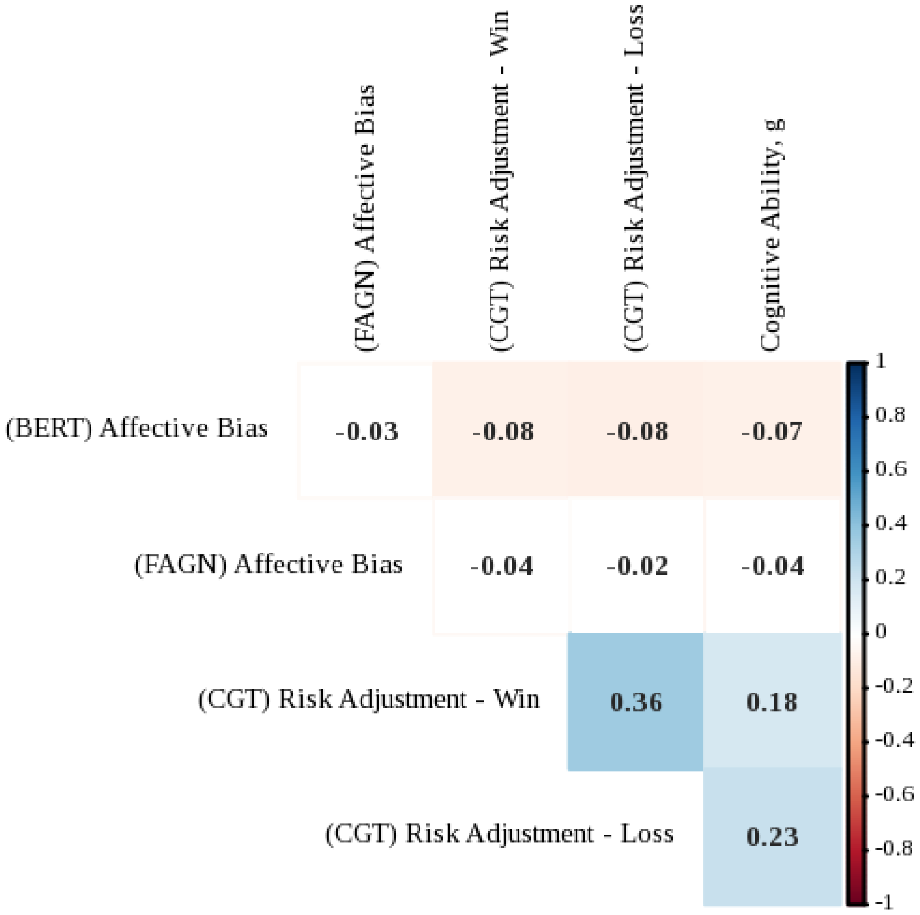

Figure S1. Pearson correlations between main affective cognitive outcome variables and *g*

BERT, Bristol Emotion Recognition Task; CGT, adapted Cambridge Gambling Task; FAGN, Face Go/No-Go

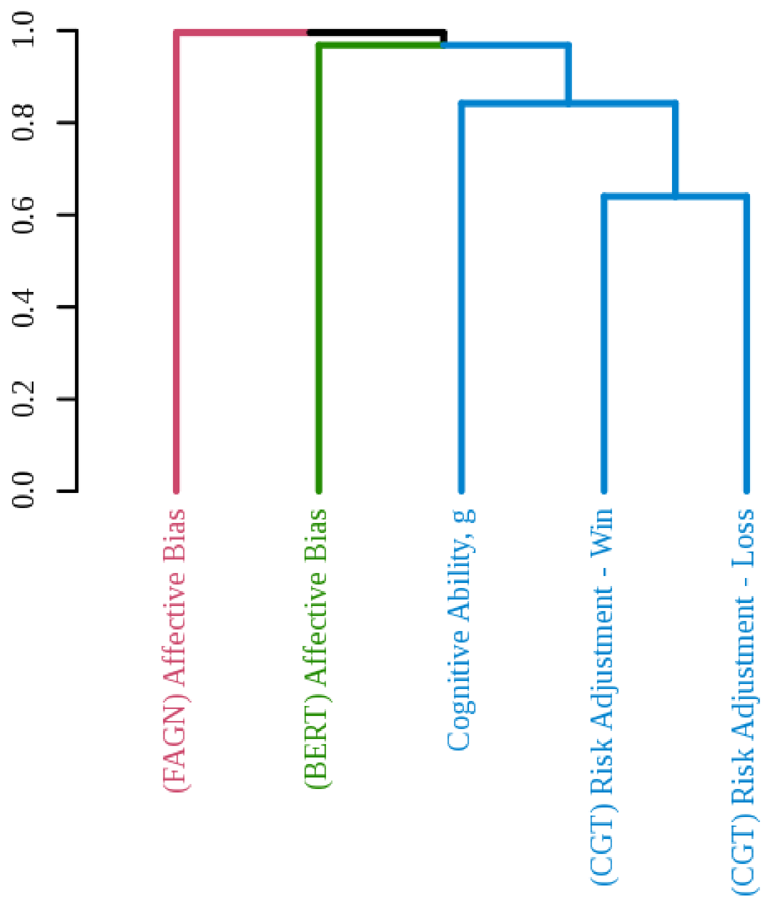

Figure S2. Hierarchical relationships between main affective cognitive outcome variables and *g*

BERT, Bristol Emotion Recognition Task; CGT, adapted Cambridge Gambling Task; FAGN, Face Go/No-Go

Statistical analysis: information on weak priors and model parameters

In all analyses, priors for the fixed effects were gaussian priors with expected value $\mu = 0$ and high variance ($V = I\times10^{10}$), which is the default for the 'MCMCglmm' algorithm. According to common implementation, non-informative priors for the residual structure (R-structure) took the shape of an inverse Wishart distribution with parameters V = 1 and nu = 0.002, which corresponds to an inverse gamma distribution with a mode very close to zero. Priors for the random structures (G-structures) also took the shape of an inverse Wishart distribution, but with parameters V = 1 and nu = 1, corresponding to an inverse gamma distribution with a mode of 0.33. This more informative prior was decided on because of a priori expectations of family-related and individual effects, following post-hoc optimisation so that the random effects in the models would pass model diagnostic tests. All 'MCMCglmm' models ran for 1×10^6 iterations with a burn-in parameter of 1×10^5 and a thinning parameter of 900, so that in accordance with recommendations, each run stored 1000 iterations. These model parameters were implemented in order to achieve sufficient model convergence with regard to all effects, while also showing an acceptable model estimation time (i.e., 5-8 hours per model when running four chains using parallel cores, with R package 'parallel').

With use of the priors and model parameters described above, all models showed reliable convergence across multiple runs, as indicated by good Gelman diagnostics (0.99-1.02 for each parameter) and sufficient mixing of four different chains; for full diagnostic information of each model see section 'additional information'. With a thinning parameter of 900, autocorrelations were low for fixed effects ($r < 0.1$), but still present in most models for family-related and individual random effects (often r = 0.2-0.6). However, given that for the purpose of the current study we only interpret the fixed effects, we considered this elevated autocorrelation of no concern to reliable interpretation. Furthermore, reducing this autocorrelation further would need a substantial increase in iterations, which would result in unacceptable model estimation times.

Statistical analysis: Detailed analyses (per task)

Detailed analysis of BERT task

First, general effects of the MDD predictors on BERT accuracy were explored by two logistic models in which emotion recognition accuracy was aggregated over all six emotions. Subsequently, emotion-specific biases were assessed with four logistic models: two models regarding accuracy of responses, and two regarding false alarm rate (i.e. that is how often the emotion was wrongly selected on trials of other emotions). These models included the main effect of emotion, the main effect of the MDD predictor (either depressive symptoms across the whole sample, or MDD-r versus controls) and the MDD by emotion interaction effect; all effects of emotion were investigated relative to the happy emotion. For each model, positive coefficients indicated better performance regarding the outcome variable; that is, higher accuracy or lower false alarm rate.

Detailed analysis of FAGN task

Further exploration of FAGN task outcome variables included the following measures of affective cognitive performance: hit/miss rate, false alarm rate and average hit trial reaction time. Hit/miss rate and false alarm rate were investigated with logistic models, in which positive coefficients reflected better performance, that is higher hit rate or lower false alarm rate. Average hit trial reaction time was investigated with Gaussian models, in which positive coefficients indicated slower reaction times, that is lower task performance.

First, general effects of each MDD predictor (either depressive symptoms across the whole sample, or MDD-r versus controls) on FAGN outcome measures were explored by six models in which each of these outcome measures were aggregated over all six conditions. Next, six additional models assessed condition-specific affective biases. These models included the main effect of condition, the main effect of the MDD predictor (either depressive symptoms across the whole sample, or MDD-r versus controls) and the MDD by condition interaction effect; all effects of condition were investigated relative to the happy/neutral condition.

Statistical analysis: sensitivity analyses regarding use of antidepressant medication

For each statistically significant effects in summary or detailed analyses, sensitivity analyses explored whether effects were related to use of antidepressant medication. For the exploration of associations with depressive symptoms, individuals with lifetime depression (MDD-r and MDD-c) were stratified into two groups based on current use of antidepressant medication (yes/no). Subsequently, the symptoms model analysis was performed twice, restricting analysis in turn to either of these groups. As such, this provided effect sizes separately for individuals with lifetime depression who used antidepressant medication at the time of assessment, and those who did not use antidepressant medication. For the exploration of group differences between MDD-r versus controls, individuals remitted from depression (MDD-r) were stratified into two groups based on current use of antidepressant medication (yes/no). Subsequently, we compared these groups (independently) with a 'clean' control group (n = 744; control participants taking antidepressant medication were excluded) using two separate models. Again, this provided effect sizes separately for those who did and those who did not use antidepressant medication at the time of assessment. In each model statistically tested coefficients, and although coefficients were not statistically compared between models, differences in effect sizes were considered to show trends regarding the effect antidepressant use on the association between depression and affective cognition.

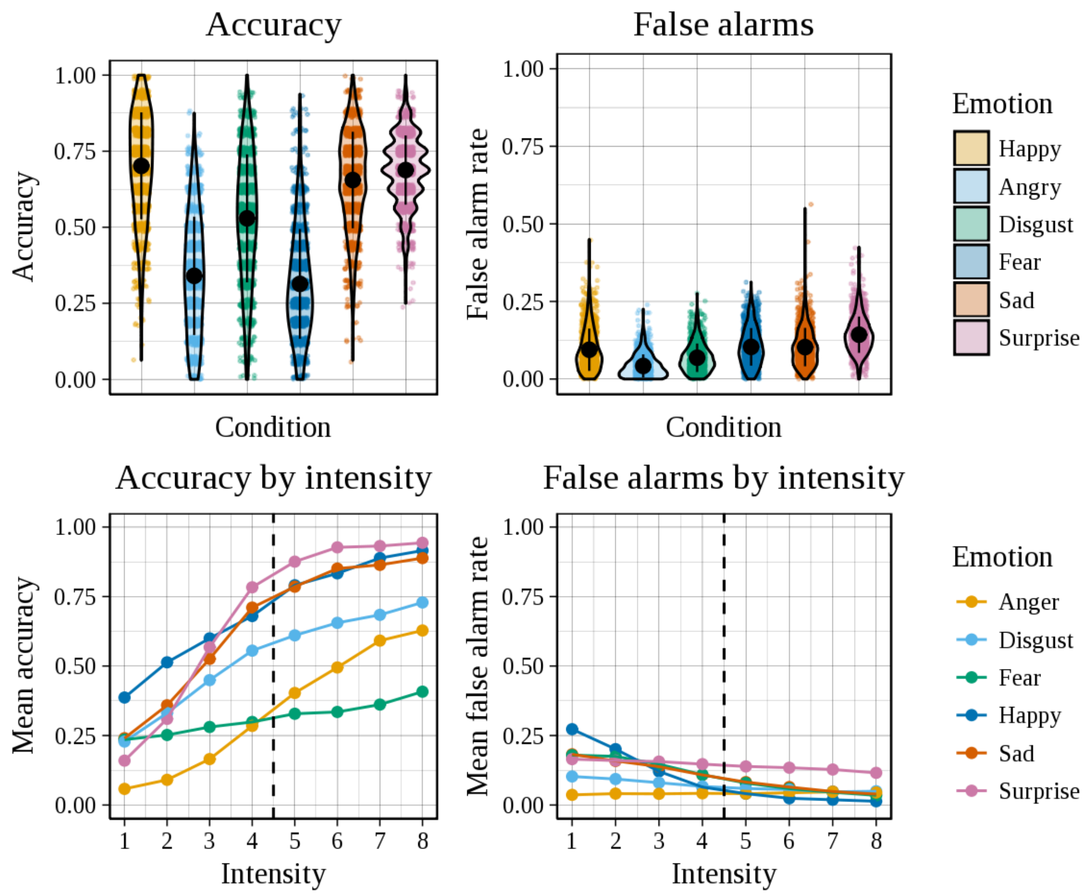

Figure S3. BERT response patterns across the entire sample

This figure shows different Bristol Emotion Recognition Task (BERT) response patterns for each emotion. In the left and right subplots, the dependent variable represents accuracy or false alarms, respectively, as a proportion of the total number of trials (i.e., 16 per emotion for accuracy, and 80 per emotion for false alarm rate). The upper subplots display the distribution of the response variables across the sample with a violin plots and jittered data points. Means and standard deviations (per emotion) are indicated by the black dots and vertical lines. The lower subplots reflect mean of the response variables across the sample as function of intensity of the emotion displayed.

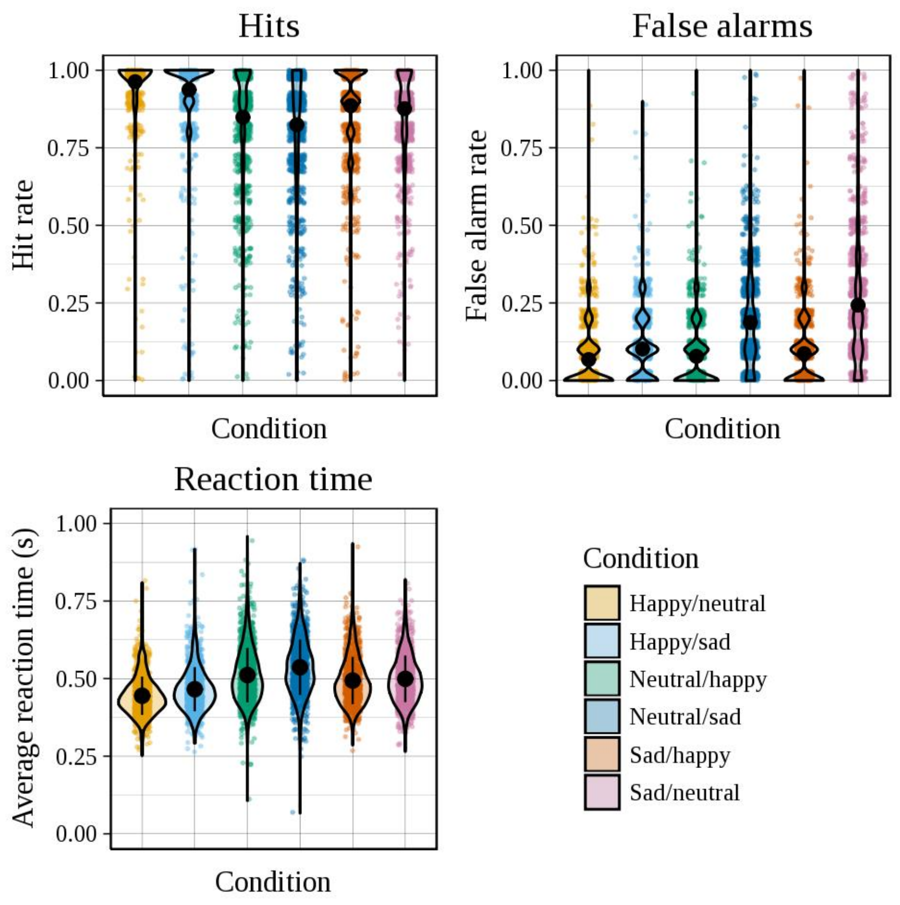

Figure S4. FAGN response patterns across the entire sample

This figure shows different Face Affective Go/No-Go (FAGN) response patterns for each condition. The dependent variable represents hits or false alarms as a proportion of the total number of trials (i.e., 10 targets per condition for hits, and 10 distractors per condition for false alarms), or average reaction time across hit trials (max. 10 per condition). The violin plots and jittered data points show the distribution of the response variables across the sample. Means (per condition) are indicated by the black dot. In the subplot showing reaction time, the black vertical lines also show the standard deviations.

Table S2. Results of detailed analyses that investigated main effects of depressive symptoms and remitted depression by aggregating affective cognitive performance measures over emotions/conditions

**BERT, accuracy**	$M_{posterior}$	**OR**	**OR 95%-CI**	***p*MCMC**
Symptoms	-0.008	0.99	[0.99, 1.00]	0.014*
MDD-r	0.015	1.02	[0.97, 1.07]	0.586
**FAGN, hit rate**	$M_{posterior}$	**OR**	**OR 95%-CI**	**pMCMC**
Symptoms	-0.037	0.96	[0.95, 0.98]	<0.001***
MDD-r	-0.095	0.91	[0.79, 1.06]	0.192
**FAGN, false alarms**	$M_{posterior}$	**OR**	**OR 95%-CI**	***p*MCMC**
Symptoms	-0.015	0.99	[0.97, 1.00]	0.052
MDD-r	0.025	1.02	[0.93, 1.12]	0.642
**FAGN, reaction time**	$M_{posterior}$		**95%-CI**	***p*MCMC**
Symptoms	0.034		[0.02, 0.05]	<0.001***
MDD-r	0.030		[-0.10,0.17]	0.684

* *p*MCMC < 0.05, ** *p*MCMC < 0.01, *** *p*MCMC < 0.001

BERT, Bristol Emotion Recognition Task; CI, credible interval; FAGN, Face Go/No-Go; MCMC, Markov Chain Monte Carlo; MDD-r, remitted from Major Depressive Disorder; OR, odds ratio.

Table S3. Results of follow-up analyses regarding the role of cognitive ability (*g*) in statistically significant associations between depressive symptoms and affective cognitive performance measures aggregated over emotions/conditions

**BERT, accuracy**	$M_{posterior}$	**%effect**	**OR**	**OR 95%-CI**	***p*MCMC**
g	0.141		1.15	[1.13, 1.17]	<0.001***
Symptoms	-0.002	24%	1.00	[0.99, 1.00]	0.522
**FAGN, hit rate**	$M_{posterior}$	**%effect**	**OR**	**OR 95%-CI**	***p*MCMC**
g	0.324		1.38	[1.30, 1.48]	<0.001***
Symptoms	-0.022	60%	0.98	[0.96, 1.00]	0.020*
**FAGN, false alarms**	$M_{posterior}$	**%effect**	**OR**	**OR 95%-CI**	***p*MCMC**
g	0.187		1.22	[1.15, 1.26]	<0.001***
Symptoms	-0.007	46%	0.99	[0.98. 1.01]	0.286
**FAGN, reaction time**	$M_{posterior}$	**%effect**		**95%-CI**	***p*MCMC**
g	-0.077			[-0.14, -0.02]	0.020*
Symptoms	0.031	90%		[0.01, 0.05]	<0.001***

* *p*MCMC < 0.05, ** *p*MCMC < 0.01, *** *p*MCMC < 0.001

BERT, Bristol Emotion Recognition Task; CI, credible interval; FAGN, Face Go/No-Go; *g*, general factor of cognitive ability; MCMC, Markov Chain Monte Carlo; OR, odds ratio.

Table S4. Results of sensitivity analyses regarding use of antidepressant medication for statistically significant associations between depressive symptoms and affective cognitive performance measures aggregated over emotions/conditions

LIFETIME MDD – ANTIDEPRESSANT USE

**BERT, accuracy (*n* = 109)**	$M_{posterior}$	**OR**	**OR 95%-CI**	***p*MCMC**
Symptoms	-0.014	0.99	[0.97, 1.00]	0.026*
**FAGN, hit rate (*n* = 106)**	$M_{posterior}$	**OR**	**OR 95%-CI**	***p*MCMC**
Symptoms	-0.036	0.96	[0.92, 1.01]	0.110
**FAGN, false alarms (*n* = 106)**	$M_{posterior}$	**OR**	**OR 95%-CI**	***p*MCMC**
Symptoms	-0.012	0.99	[0.97, 1.01]	0.350
**FAGN, reaction time (*n* = 106)**	$M_{posterior}$		**95%-CI**	***p*MCMC**
Symptoms	0.027		[-0.01, 0.06],	0.112

LIFETIME MDD – NO ANTIDEPRESSANT USE

**BERT, accuracy (*n* = 214)**	$M_{posterior}$	**OR**	**OR 95%-CI**	***p*MCMC**
Symptoms	-0.008	0.99	[0.98, 1.01]	0.280
**FAGN, hit rate (*n* = 214)**	$M_{posterior}$	**OR**	**OR 95%-CI**	***p*MCMC**
Symptoms	-0.060	0.94	[0.91, 0.98]	0.004**
**FAGN, false alarms (*n* = 214)**	$M_{posterior}$	**OR**	**OR 95%-CI**	***p*MCMC**
Symptoms	-0.025	0.98	[0.95, 1.00]	0.080
**FAGN, reaction time (*n* = 214)**	$M_{posterior}$		**95%-CI**	***p*MCMC**
Symptoms	0.062		[0.03, 0.10]	0.002**

* *p*MCMC < 0.05, ** *p*MCMC < 0.01, *** *p*MCMC < 0.001

BERT, Bristol Emotion Recognition Task; CI, credible interval; FAGN, Face Go/No-Go; MCMC, Markov Chain Monte Carlo; MDD, Major Depressive Disorder; OR, odds ratio.

Table S5. Results of detailed analyses regarding emotion/condition-specific associations between depressive symptoms and affective cognitive performance

**BERT, accuracy**	$M_{posterior}$	**OR**	**OR 95%-CI**	***p*MCMC**
Symptoms (ref. Happy)	-0.019	0.98	[0.97, 1.00]	0.006**
Symptoms:Angry	0.026	1.03	[1.01, 1.05]	0.014*
Symptoms:Disgust	0.003	1.00	[0.98, 1.02]	0.740
Symptoms:Fear	0.018	1.02	[1.00, 1.04]	0.078
Symptoms:Sad	0.003	1.00	[0.98, 1.02]	0.758
Symptoms:Surprise	0.004	1.00	[0.99, 1.02]	0.630
**BERT, false alarms**	$M_{posterior}$	**OR**	**OR 95%-CI**	***p*MCMC**
Symptoms (ref. Happy)	0.007	1.01	[0.99, 1.02]	0.252
Symptoms:Angry	-0.038	0.96	[0.94, 0.98]	<0.001***
Symptoms:Disgust	-0.024	0.98	[0.96, 0.99]	0.008**
Symptoms:Fear	-0.011	0.99	[0.97, 1.01]	0.218
Symptoms:Sad	-0.006	0.99	[0.98, 1.01]	0.496
Symptoms:Surprise	-0.011	0.99	[0.97, 1.01]	0.214
**FAGN, hit rate**	$M_{posterior}$	**OR**	**OR 95%-CI**	***p*MCMC**
Symptoms (ref. Happy/neutral)	-0.072	0.93	[0.89, 0.98]	0.004**
Symptoms:Happy/sad	0.030	1.03	[0.98, 1.10]	0.340
Symptoms:Neutral/happy	0.011	1.01	[0.96, 1.07]	0.632
Symptoms:Neutral/sad	0.011	1.01	[0.96, 1.06]	0.658
Symptoms:Sad/happy	0.046	1.05	[1.00, 1.11]	0.096
Symptoms:Sad/neutral	0.055	1.06	[1.00, 1.11]	0.058
**FAGN, false alarms**	$M_{posterior}$	**OR**	**OR 95%-CI**	***p*MCMC**
Symptoms (ref. Happy/neutral)	-0.024	0.98	[0.95, 1.01]	0.140
Symptoms:Happy/sad	0.012	1.01	[0.98, 1.05]	0.490
Symptoms:Neutral/happy	-0.027	0.97	[0.94, 1.01]	0.164
Symptoms:Neutral/sad	0.012	1.01	[0.98, 1.04]	0.476
Symptoms:Sad/happy	-0.008	0.99	[0.96, 1.03]	0.688
Symptoms:Sad/neutral	0.028	1.03	[1.00, 1.07]	0.104
**FAGN, reaction time**	$M_{posterior}$		**95% CI**	***p*MCMC**
Symptoms (ref. Happy/neutral)	0.014		[0.00, 0.03]	0.080
Symptoms:Happy/sad	-0.008		[-0.02, 0.01]	0.296
Symptoms:Neutral/happy	-0.026		[-0.04, -0.01]	0.002**

Symptoms:Neutral/sad	-0.015	[-0.03, 0.00]	0.068
Symptoms:Sad/happy	-0.015	[-0.03, 0.00]	0.072
Symptoms:Sad/neutral	-0.020	[-0.04, -0.01]	0.018*

* *p*MCMC < 0.05, ** *p*MCMC < 0.01, *** *p*MCMC < 0.001

BERT, Bristol Emotion Recognition Task; CI, credible interval; FAGN, Face Go/No-Go; MCMC, Markov Chain Monte Carlo; OR, odds ratio; ref., reference emotion/condition.

Table S6. Results of detailed analyses regarding emotion/condition-specific associations between remitted depression and affective cognition

**BERT, accuracy**	***M*posterior**	**OR**	**OR 95%-CI**	***p*MCMC**
MDD-r (ref. Happy)	0.030	1.03	[0.93, 1.17]	0.608
MDD-r:Angry	0.100	1.11	[0.96, 1.28]	0.154
MDD-r:Disgust	-0.025	0.98	[0.85, 1.14]	0.738
MDD-r:Fear	-0.011	0.99	[0.86, 1.14]	0.878
MDD-r:Sad	-0.049	0.95	[0.83, 1.12]	0.518
MDD-r:Surprise	-0.099	0.91	[0.77, 1.03]	0.158
**BERT, false alarms**	***M*posterior**	**OR**	**OR 95%-CI**	***p*MCMC**
MDD-r (ref. Happy)	-0.059	0.94	[0.85, 1.05]	0.248
MDD-r:Angry	-0.051	0.95	[0.82, 1.11]	0.512
MDD-r:Disgust	0.027	1.03	[0.89, 1.17]	0.726
MDD-r:Fear	0.114	1.12	[0.98, 1.28]	0.098
MDD-r:Sad	0.150	1.16	[1.00, 1.32]	0.032*
MDD-r:Surprise	0.094	1.10	[0.97, 1.26]	0.176
**FAGN, hit rate**	***M*posterior**	**OR**	**OR 95%-CI**	***p*MCMC**
MDD-r (ref. Happy/neutral)	-0.082	0.92	[0.63, 1.39]	0.686
MDD-r:Happy/sad	-0.211	0.81	[0.50, 1.28]	0.372
MDD-r:Neutral/happy	0.062	1.06	[0.69, 1.63]	0.782
MDD-r:Neutral/sad	-0.052	0.95	[0.61, 1.43]	0.830
MDD-r:Sad/happy	0.013	1.01	[0.65, 1.57]	0.982
MDD-r:Sad/neutral	-0.007	0.99	[0.66, 1.54]	0.958
**FAGN, false alarms**	***M*posterior**	**OR**	**OR 95%-CI**	***p*MCMC**
MDD-r (ref. Happy/neutral)	-0.124	0.88	[0.71, 1.09]	0.288
MDD-r:Happy/sad	0.208	1.23	[0.89, 1.56]	0.136
MDD-r:Neutral/happy	0.178	1.19	[0.90, 1.62]	0.272
MDD-r:Neutral/sad	0.028	1.03	[0.77, 1.31]	0.814
MDD-r:Sad/happy	0.032	1.03	[0.76, 1.36]	0.858
MDD-r:Sad/neutral	0.284	1.33	[1.02, 1.70]	0.036*
**FAGN, reaction time**	***M*posterior**		**95% CI**	***p*MCMC**
MDD-r (ref. Happy/neutral)	0.102		[-0.02, 0.24]	0.130
MDD-r:Happy/sad	-0.074		[-0.20, 0.05]	0.262
MDD-r:Neutral/happy	-0.102		[-0.23, 0.02]	0.116

MDD-r:Neutral/sad	-0.034	[-0.15, 0.12]	0.620
MDD-r:Sad/happy	-0.133	[-0.27, 0.00]	0.050*
MDD-r:Sad/neutral	-0.102	[-0.23, 0.03]	0.134

* *p*MCMC < 0.05, ** *p*MCMC < 0.01, *** *p*MCMC < 0.001

BERT, Bristol Emotion Recognition Task; CI, credible interval; FAGN, Face Go/No-Go; MCMC, Markov Chain Monte Carlo; MDD-r, remitted from Major Depressive Disorder; OR, odds ratio.

Table S7. Results of follow-up analyses regarding the role of cognitive ability (*g*) in statistically significant emotion/condition-specific associations between depressive symptoms and affective cognition

**CGT, risk adjustment win**	$M_{posterior}$	**%effect**		**95%-CI**	***p*MCMC**
g	0.174			[0.12, 0.23]	<0.001***
Symptoms	-0.011	59%		[-0.03, 0.01]	0.174
**BERT, accuracy**	$M_{posterior}$	**%effect**	**OR**	**OR 95%-CI**	***p*MCMC**
g	0.166		1.18	[1.15, 1.21]	<0.001***
Symptoms (ref. Happy)	-0.012	62%	0.99	[0.97, 1.00]	0.104
Symptoms:Angry	0.025	99%	1.03	[1.01, 1.04]	<0.001***
**BERT, false alarms**	$M_{posterior}$	**%effect**	**OR**	**OR 95%-CI**	***p*MCMC**
g	0.087		1.09	[1.07, 1.11]	<0.001***
Symptoms:Angry	-0.038	99%	0.96	[0.95, 0.98]	<0.001***
Symptoms:Disgust	-0.023	98%	0.98	[0.96, 0.99]	0.004**
**FAGN, hit rate**	$M_{posterior}$	**%effect**	**OR**	**OR 95%-CI**	***p*MCMC**
g	0.393		1.48	[1.38, 1.60]	<0.001***
Symptoms (ref. Happy/neutral)	-0.055	76%	0.95	[0.90, 0.99]	0.018*
**FAGN, reaction time**	$M_{posterior}$	**%effect**		**95%-CI**	***p*MCMC**
g	-0.031			[-0.08, 0.01]	0.160
Symptoms:Neutral/happy	-0.026	101%		[-0.04, -0.01]	0.002**
Symptoms:Sad/neutral	-0.020	98%		[-0.04, -0.01]	0.018*

* pMCMC < 0.05, ** pMCMC < 0.01, *** pMCMC < 0.001

BERT, Bristol Emotion Recognition Task; CGT, adapted Cambridge Gambling Task; CI, credible interval; FAGN, Face Go/No-Go; *g*, general factor of cognitive ability; MCMC, Markov Chain Monte Carlo; OR, odds ratio; ref., reference emotion/condition.

Table S8. Results of follow-up analyses regarding the role of cognitive ability (*g*) in statistically significant emotion/condition-specific associations between remitted depression and affective cognition

**BERT, false alarms**	$M_{posterior}$	**%effect**	**OR**	**OR 95%-CI**	***p*MCMC**
g	0.086		1.09	[1.07, 1.11]	<0.001***
MDD-r:Sad	0.157	104%	1.17	[1.01, 1.32]	0.024*
**FAGN, false alarms**	$M_{posterior}$	**%effect**	**OR**	**OR 95%-CI**	***p*MCMC**
g	0.218		1.24	[1.18, 1.30]	<0.001***
MDD-r:Sad/neutral	0.288	101%	1.33	[1.05, 1.71]	0.012*
**FAGN, reaction time**	$M_{posterior}$	**%effect**		**95%-CI**	***p*MCMC**
g	-0.033			[-0.08, 0.01]	0.142
MDD-r:Sad/happy	-0.132	99%		[-0.26, -0.01]	0.034*

* *p*MCMC < 0.05, ** *p*MCMC < 0.01, *** *p*MCMC<0.001

BERT, Bristol Emotion Recognition Task; CI, credible interval; FAGN, Face Go/No-Go; *g*, general factor of cognitive ability; MCMC, Markov Chain Monte Carlo; MDD-r, remitted from Major Depressive Disorder; OR, odds ratio.

Table S9. Results of sensitivity analyses regarding subclinical symptoms for statistically significant emotion/condition-specific associations between remitted depression and affective cognition

**BERT, false alarms**	**$M_{posterior}$**	**OR**	**OR 95%-CI**	***p*MCMC**
Symptoms:Sad	-0.019	0.98	[0.95, 1.01]	0.248
**FAGN, false alarms**	**$M_{posterior}$**	**OR**	**OR 95%-CI**	***p*MCMC**
Symptoms:Sad/neutral	0.036	1.04	[0.98, 1.09]	0.186
**FAGN, reaction time**	**$M_{posterior}$**		**95% CI**	***p*MCMC**
Symptoms:Sad/happy	-0.013		[-0.04, 0.02]	0.352

* *p*MCMC < 0.05, ** *p*MCMC < 0.01, *** *p*MCMC < 0.001

BERT, Bristol Emotion Recognition Task; CI, credible interval; FAGN, Face Go/No-Go; MCMC, Markov Chain Monte Carlo; OR, odds ratio.

Table S10. Results of sensitivity analyses regarding use of antidepressant medication for statistically significant emotion/condition-specific associations between depressive symptoms and affective cognition

LIFETIME MDD – ANTIDEPRESSANT USE

**CGT, RA win (*n* = 111)**	$M_{posterior}$		**95%-CI**	***p*MCMC**
Symptoms	-0.050		[-0.09, -0.02]	0.004**
**BERT, accuracy (*n* = 109)**	$M_{posterior}$	**OR**	**OR 95%-CI**	***p*MCMC**
Symptoms (ref. Happy)	-0.044	0.96	[0.93, 0.98]	0.002**
Symptoms:Angry	0.035	1.04	[1.00, 1.07]	0.044*
**BERT, false alarms (*n* = 109)**	$M_{posterior}$	**OR**	**OR 95%-CI**	***p*MCMC**
Symptoms:Angry	-0.057	0.94	[0.91, 0.98]	0.006**
Symptoms:Disgust	-0.052	0.95	[0.92, 0.98]	0.004**
**FAGN, hit rate (*n* = 106)**	$M_{posterior}$	**OR**	**OR 95%-CI**	***p*MCMC**
Symptoms (ref. Happy/neutral)	-0.053	0.95	[0.86, 1.08]	0.384
**FAGN, reaction time (*n* = 106)**	$M_{posterior}$		**95%-CI**	***p*MCMC**
Symptoms:Neutral/happy	-0.013		[-0.05, 0.02]	0.444
Symptoms:Sad/neutral	-0.008		[-0.04, 0.02]	0.604

LIFETIME MDD – NO ANTIDEPRESSANT USE

**CGT, RA win (*n* = 214)**	$M_{posterior}$		**95%-CI**	***p*MCMC**
Symptoms	0.015		[-0.02, 0.05]	0.418
**BERT, accuracy (*n* = 214)**	$M_{posterior}$	**OR**	**OR 95%-CI**	***p*MCMC**
Symptoms (ref. Happy)	-0.030	0.97	[0.94, 1.00]	0.056
Symptoms:Angry	0.049	1.05	[1.01, 1.09]	0.008**
**BERT, false alarms (*n* = 214)**	$M_{posterior}$	**OR**	**OR 95%-CI**	***p*MCMC**
Symptoms:Angry	-0.042	0.96	[0.92, 0.99]	0.032*
Symptoms:Disgust	-0.027	0.97	[0.94, 1.01]	0.126
**FAGN, hit rate (*n* = 214)**	$M_{posterior}$	**OR**	**OR 95%-CI**	***p*MCMC**
Symptoms (ref. Happy/neutral)	-0.075	0.93	[0.84, 1.03]	0.126
**FAGN, reaction time (*n* = 214)**	$M_{posterior}$		**95%-CI**	***p*MCMC**
Symptoms:Neutral/happy	-0.025		[-0.06, 0.01]	0.156
Symptoms:Sad/neutral	-0.038		[-0.07, 0.00]	0.028*

* pMCMC < 0.05, ** pMCMC < 0.01, *** pMCMC < 0.001

BERT, Bristol Emotion Recognition Task; CGT, adapted Cambridge Gambling Task; CI, credible interval; FAGN, Face Go/No-Go; MCMC, Markov Chain Monte Carlo; MDD, Major Depressive Disorder; OR, odds ratio; RA, risk adjustment.

Table S11. Results of sensitivity analyses regarding use of antidepressant medication for statistically significant emotion/condition-specific associations between remitted depression and affective cognition

MDD-R – ANTIDEPRESSANT USE

**BERT, false alarms (*n* = 84)**	$M_{posterior}$	**OR**	**OR 95%-CI**	***p*MCMC**
MDD-r:Sad	0.240	1.27	[1.02, 1.60]	0.042*
**FAGN, false alarms (*n* = 83)**	$M_{posterior}$	**OR**	**OR 95%-CI**	***p*MCMC**
MDD-r:Sad/neutral	0.204	1.23	[0.81, 1.85]	0.352
**FAGN, reaction time (*n* = 83)**	$M_{posterior}$		**95% CI**	***p*MCMC**
MDD-r:Sad/happy	-0.308		[-0.51, -0.09]	0.008**

MDD-R – NO ANTIDEPRESSANT USE

**BERT, false alarms (*n* = 197)**	$M_{posterior}$	**OR**	**OR 95%-CI**	***p*MCMC**
MDD-r:Sad	0.116	1.12	[0.96, 1.31]	0.172
**FAGN, false alarms (*n* = 197)**	$M_{posterior}$	**OR**	**OR 95%-CI**	***p*MCMC**
MDD-r:Sad/neutral	0.319	1.38	[1.02, 1.83]	0.036*
**FAGN, reaction time (*n* = 197)**	$M_{posterior}$		**95% CI**	**pMCMC**
MDD-r:Sad/happy	-0.063		[-0.22, 0.08]	0.396

* *p*MCMC < 0.05, ** *p*MCMC < 0.01, *** *p*MCMC < 0.001

BERT, Bristol Emotion Recognition Task; CI, credible interval; FAGN, Face Go/No-Go; MCMC, Markov Chain Monte Carlo; MDD-r, remitted from Major Depressive Disorder; OR, odds ratio.

Additional information

The following additional information will be available online via **https://osf.io/a2957/**

- Analysis scripts (R code)
- Model diagnostic information regarding convergence and mixing of chains (PDF files)
- Additional results (Excel files)
    - Full model outputs, including mean posterior estimates for covariates
    - Model outputs that include assessment site as additional covariate (only for main analyses)
